# Better treatment outcomes with aripiprazole long-acting injection in community and incarcerated patients with serious mental illness

**DOI:** 10.3389/fpsyt.2025.1499400

**Published:** 2025-08-08

**Authors:** Erasmia I. Koiliari, Ioannis Mouzas, Georgios Alevizopoulos, Otto Lesch, Henriette Walter, Emmanouil L. Pasparakis

**Affiliations:** ^1^ Laboratory of Alcohology, Department of Pathology, Medical School of Crete, University of Crete, Herakleion, Greece; ^2^ Department of Psychiatry, General Hospital of Agios Nikolaos, Agios Nikolaos, Greece; ^3^ Department of Psychiatry, Agioi Anargyroi Hospital, National and Kapodistrian University of Athens, Athens, Greece; ^4^ Department of Social Psychiatry, Medical University of Vienna, Vienna, Austria

**Keywords:** aripiprazole, long -acting treatment, serious mental illness (SMI), psychosis, quality of life, hospitalizations, functionality, substance use disorder (SUD)

## Abstract

**Importance:**

Aripiprazole, a partial D2 receptor agonist, is proposed to enhance prefrontal cortex (PFC) dopamine function, improving working memory and GABA transmission, which supports social functioning. Long-acting injectable (LAI) antipsychotics are known to improve patient adherence, leading to enhanced long-term effects on behavioral outcomes.

**Objective:**

To evaluate whether aripiprazole LAI treatment improves general functioning, quality of life, and reduces hospitalizations in psychotic patients, both in community settings and within incarcerated populations.

**Design, settings, and participants:**

The study included 55 patients, with 34 from the community and 21 incarcerated at a prison in Southeastern Greece (Neapolis). The World Health Organization Quality of Life Brief Version (WHOQOL-BREF) and the Clinical Global Impression-Severity (CGI-S) scale were used to assess outcomes. Comparisons were made between pre-treatment and post-treatment periods, with a minimum follow-up of six months.

**Results:**

• Demographics: Community patients (70.6% male) included 44.1% with paranoid schizophrenia. Incarcerated patients (all male) had an F29.0 diagnosis, with 57.1% exhibiting Cluster B personality disorder and all reporting psychoactive substance use.

• Hospitalizations: Community patients’ hospitalizations decreased from 1.4 to 0.1 over six months (p=0.001). Incarcerated patients’ hospitalizations dropped from 0.6 to 0.0 (p=0.066), with no significant intergroup difference (p=0.150).

• CGI-S: Community patients’ scores improved from 6.0 to 3.9 (p<0.001). Incarcerated patients’ scores improved from 5.3 to 3.2 (p<0.001), with no significant difference between groups (p=0.814).

• Quality of Life: Community patients’ scores rose from 0.5 to 3.0 (p<0.001), while incarcerated patients’ scores also increased significantly (p<0.001).

**Conclusions:**

This study of 34 community and 21 incarcerated patients revealed significant demographic and medical history differences. Both groups experienced reduced hospitalizations and improvements in CGI-S scores and quality of life following aripiprazole LAI administration. Community patients showed a greater reduction in hospitalizations, while clinical and quality-of-life improvements were comparable across groups.

## Introduction

Non-adherence to antipsychotic medication is a primary reason for treatment inefficacy in psychotic patients, particularly those with co-occurring substance use disorders (SUD) or alcohol use disorders (AUD). Poor adherence increases relapse risk, hospitalization rates, treatment costs, and the likelihood of legal issues, perpetuating a “revolving door” cycle of hospitalization and incarceration. In Greece, the impending implementation of Law 5129/2024, effective February 1, 2025, will integrate psychiatric services with the penitentiary system, prompting this study to assess aripiprazole LAI benefits for community and incarcerated patients in Eastern Crete. The findings aim to inform health policy, optimize resource allocation, and support psychiatric reform. Long-acting injectable (LAI) antipsychotics, such as aripiprazole once-monthly monohydrate (AOM) and aripiprazole lauroxil, address non-adherence by reducing daily dosing needs. AOM, approved for schizophrenia since 2012 and bipolar disorder since 2017, and aripiprazole lauroxil, approved for schizophrenia, offer sustained treatment options (1). A new formulation, Ari 2MRTU 960, administered bimonthly, is under investigation for schizophrenia and bipolar I disorder (2). As a third-generation antipsychotic, aripiprazole acts as a partial D2 and 5-HT1A agonist and 5-HT2A antagonist, approved by the FDA in 2002 (1). Its unique mechanism (3) may reduce hyperdopaminergic activity in the mesolimbic system (antipsychotic effect) while enhancing hypodopaminergic activity in the prefrontal cortex, potentially alleviating negative symptoms and cognitive deficits (4–6). Partial 5-HT1A agonism may also provide anxiolytic benefits (7). Studies, including a 2015 trial in the UK and Canada, demonstrated aripiprazole’s ability to enhance dorsolateral prefrontal cortex (DLPFC) activation during working memory tasks, suggesting improved processing speed (8).

## Methods

The study enrolled 55 patients: 34 from the community (15 with schizophrenia [F20.0, ICD-10], 19 with unspecified psychosis [F29.0, ICD-10]; 35.3% with AUD, 26.5% with cannabis use disorder) and 21 incarcerated males (all with F29.0; 90.5% with AUD, 95.2% with cannabis use disorder) (**Table 1**). Ethical approval was granted by the General Hospital of Agios Nikolaos (decision 514/19-07-2023) and the Ministry of Public Order (Prot. No. 10456/10-04-2023). Participants were assessed using the WHOQOL-BREF and CGI-S scales, with outcomes compared pre- and post-aripiprazole LAI treatment over a minimum six-month period.

**Table 1:** Patient demographics and medical history.

		Sample of patients				X2 test p-value
		Patients in the community		Confined patients		
		N	%	N	%	
SEX	Male	24	70.6	21	100.0	**0.006**
	Female	10	29.4	0	.0	
ICD-10 DIAGNOSIS	F20.0 ICD-10	15	44.1	0	.0	**<0.001**
	F29.0 ICD-10	19	55.9	21	100.0	
TRAUMATIC BRAIN INJURY	No	31	91.2	21	100.0	0.162
	YES	3	8.8	0	.0	
MENTAL RETARDATION- DISCOUNT	NO - WITHIN NORMAL	18	52.9	17	81.0	0.379
	PRIMARY LIMITATION INTELLIGENCE PHYSIOLOGICAL- PATHOLOGICAL	4	11.8	3	14.3	
	PRIMARY MILD MENTAL RETARDATION	1	2.9	1	4.8	
	PRIMARY MODERATE MENTAL RETARDATION	1	2.9	0	.0	
	PRIMARY SEVERE MENTAL RETARDATION	1	2.9	0	.0	
	SECONDARY MENTAL DISCOUNT DUE TO CEC	2	5.9	0	.0	
	MILD MENTAL RECEPTION SECONDARY TO PSYCHOSIS	4	11.8	0	.0	
	SECONDARY MODERATE MENTAL IMPAIRMENT DUE TO PSYCHOSIS	2	5.9	0	.0	
	SECONDARY SEVERE MENTAL INTELLIGENCE DUE TO PSYCHOSIS	1	2.9	0	.0	
PERSONALITY TYPOLOGY	CLUSTER B -DRAMA TYPE	2	5.9	12	57.1	**<0.001**
	CLUSTER C- ANXIETY TYPE	32	94.1	9	42.9	
ALCOHOL USE DISORDER	No	22	64.7	2	9.5	**<0.001**
	YES	12	35.3	19	90.5	
COCAINE	No	32	94.1	4	19.0	**<0.001**
	YES	2	5.9	17	81.0	
CANNABIS	No	25	73.5	1	4.8	**<0.001**
	YES	9	26.5	20	95.2	
HEROIN	No	34	100.0	18	85.7	**0.023**
	YES	0	.0	3	14.3	
BENZODIAZEPINES & OTHER MEDICINAL SUBSTANCES	No	33	97.1	2	9.5	**<0.001**
	YES	1	2.9	19	90.5	
OTHER PSYCHOTRONIC SUBSTANCE	No	32	94.1	11	52.4	**<0.001**
	YES	2	5.9	10	47.6	
EVEN A PSYCHOTRONIC SUBSTANCE	No	19	55.9	0	.0	**<0.001**
	YES	15	34.1	21	100.0	
Marital status	He lives with a partner-wife- friends	10	29.4	3	14.3	**<0.001**
	He lives with parents	16	47.1	2	9.5	
	He lives alone	7	20.6	1	4.8	
	He lives in a unit	1	2.9	0	.0	
	He lives with fellow inmates in a penitentiary	0	.0	15	71.4	
EMPLOYMENT STATUS	Incapacity for work due to psychosis	18	52.9	3	14.3	**<0.001**
	Work disability independent of psychosis	3	8.8	0	.0	
	Part time job	5	14.7	2	9.5	
	Full time	6	17.6	3	14.3	
	He doesn’t work by choice	2	5.9	0	.0	
	He is not working, as he is an inmate in a Penitentiary	0	.0	13	61.9	
	Total	34	100.0	21	100.0	

Patients in the community and incarcerated patients in a Penitentiary of Greece (N=34 and N=21 respectively)

With the bold, values statistically significant.

In blue, patients in the community, in yellow-orange, patients in the penitentiary system.

### Statistical analysis

Categorical data (e.g., gender) were analyzed with frequency tables, while continuous (e.g., age) and ordinal data (e.g., CGI-S) were reported as means and standard deviations. The χ² test compared sociodemographic and medical history differences (except age), and the Mann-Whitney U test assessed age, hospitalizations, CGI-S, and quality-of-life differences between groups. The Wilcoxon signed-rank test evaluated pre- and post-treatment changes within groups, with a significance level of α=0.05.

### Results

- Community Patients (n=34): 70.6% male, mean age 42.3 ± 11.9 years, 44.1% with F20.0, 94% with Cluster C personality, 34.1% with psychoactive substance use, mean treatment duration 23.2 ± 18.3 months.
- Incarcerated Patients (n=21): All male, mean age 37.6 ± 7.7 years, all with F29.0, 57.1% with Cluster B personality, 100% with psychoactive substance use, mean treatment duration 14.5 ± 11.3 months.
- Significant differences were noted between groups in most demographic and medical history parameters, except for traumatic brain injury, mental retardation, age, and treatment duration.

### Number of Hospitalizations

**Figure 1:**
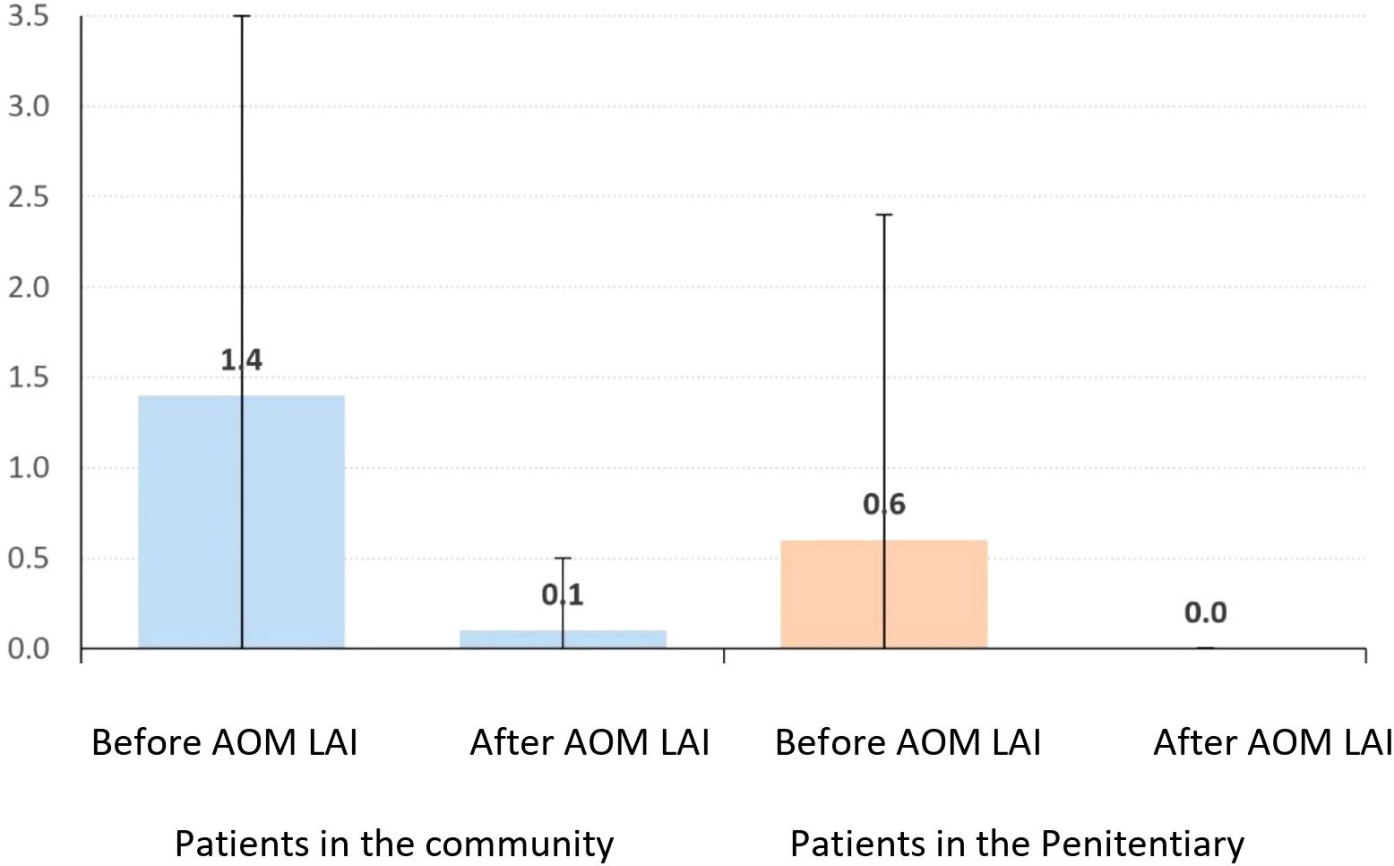
Average (+ deviation standard) of the hospitalizations before and after AOM LAI.

Community patients’ hospitalizations decreased from 1.4 ± 2.1 to 0.1 ± 0.4 (p=0.001), a mean reduction of 1.3 ± 2.1. Incarcerated patients’ hospitalizations fell from 0.6 ± 1.8 to 0.0 ± 0.0 (p=0.066), a mean reduction of 0.6 ± 1.8, with no significant intergroup difference (p=0.150) (**Tables 2**, **3**; **Figure 1**).

**Table 2:** Total hospitalizations, CGI-S scale (1=Normal, 2=Borderly ill, 3=Mildly ill, 4=Moderately ill, 5=Significantly ill, 6=Severely ill, 7=Among the most severe cases), Quality of Life scale (0=very bad, 1=bad, 2=neither good-nor bad, 3=good, 4=very good) before AOM LAI.

	Sample of patients						Mann- Whitney U Test p- value
	Patients in the community			Patients in the Penitentiary			
	Average	Typ. deviation	N	Average	Typ. Deviation	N	
Total hospitalizations before AOM LAI	1.4	2.1	34	0.6	1.8	21	0.056
CGI-S - 6 months before AOM LAI	6.0	0.9	34	5.3	0.8	21	0.006
Quality of life - 6 months before AOM LAI	0.5	0.6	34	0.9	0.6	21	0.028

In blue, patients in the community, in yellow-orange, patients in the penitentiary system.

**Table 3:** Total hospitalizations before and after AOM LAI.

	Sample of patients					
	Patients in the community			Patients in the Penitentiary		
	Average	Standard deviation	N	Average	Standard deviation	N
Total hospitalizations before AOM LAI	1.4	2.1	34	0.6	1.8	21
Total hospitalizations after AOM LAI	0.1	0.4	34	0.0	0.0	20

In blue, patients in the community, in yellow-orange, patients in the penitentiary system.

### CGI-S

**Figure 2:**
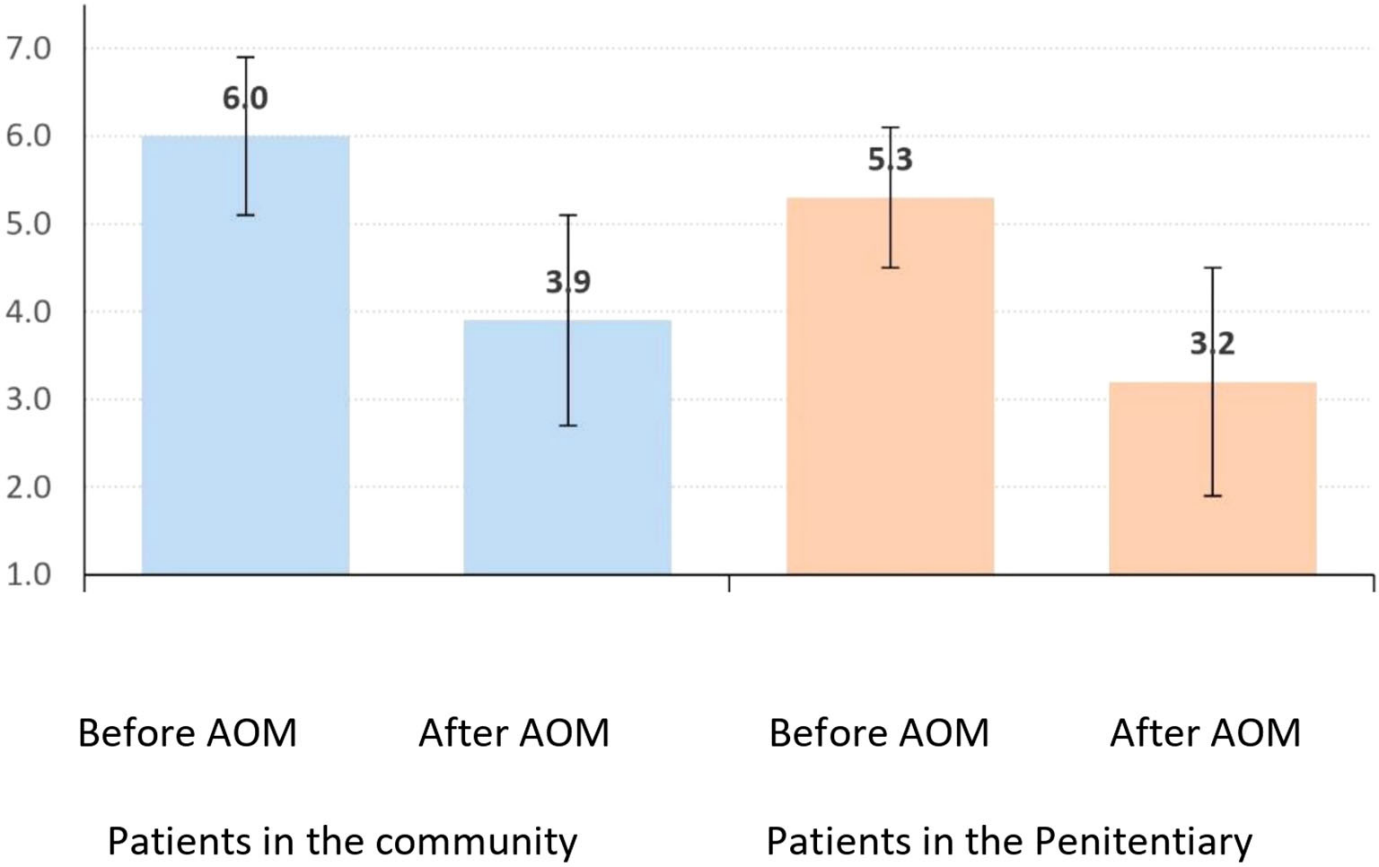
Average (+ deviation standard) of the CGI-Scale before and after AOM LAI.

Community patients’ CGI-S scores improved from 6.0 ± 0.9 to 3.9 ± 1.2 (p<0.001), a mean reduction of 2.2 ± 1.4. Incarcerated patients’ scores improved from 5.3 ± 0.8 to 3.2 ± 1.3 (p<0.001), a mean reduction of 2.1 ± 1.2, with no significant intergroup difference (p=0.814) (**Table 4**; **Figure 2**).

**Table 4:** CGI-S scale before and after AOM LAI (1=Normal, 2=Borderly ill, 3=Mildly ill, 4=Moderately ill, 5=Significantly ill, 6=Severely ill, 7=Among the most severe cases).

	Sample of patients					
	Patients in the community			Patients in the Penitentiary		
	Average	Standard deviation	N	Average	Standard Deviation	N
CGI-S - 6 months before AOM LAI	6.0	0.9	34	5.3	0.8	21
CGI-S - 6 months after AOM LAI	3.9	1.2	32	3.2	1.3	21

In blue, patients in the community, in yellow-orange, patients in the penitentiary system.

### Quality of life

**Figure 3:**
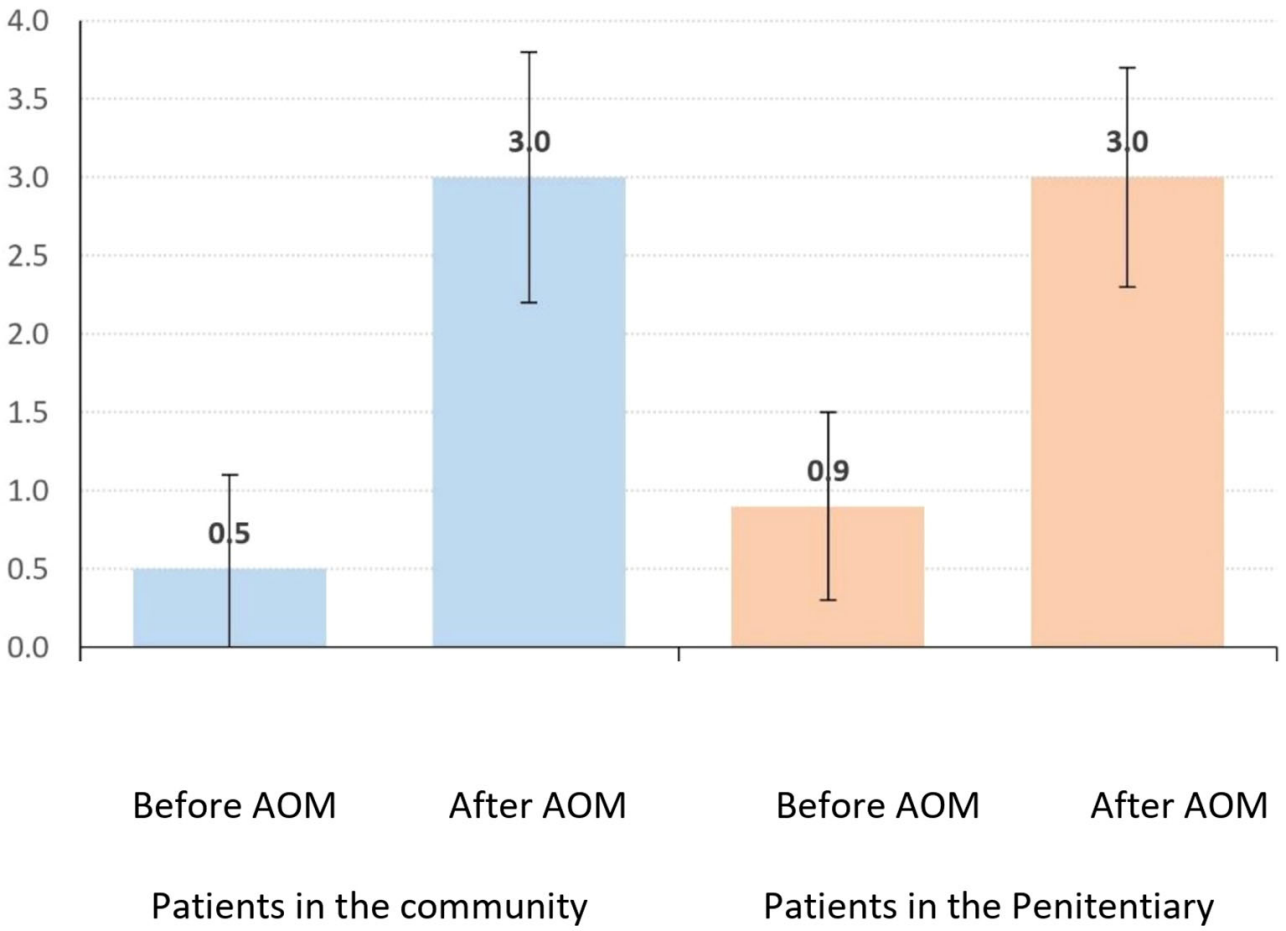
Average (+ Standard deviation) of the Quality-of-life scale before and after AOM LAI.

Community patients’ quality-of-life scores increased from 0.5 ± 0.6 to 3.0 ± 0.8 (p<0.001), a mean gain of 2.5 ± 1.0. Incarcerated patients’ scores rose from 0.9 ± 0.6 to 3.0 ± 0.7 (p<0.001), a mean gain of 2.1 ± 0.9, with no significant intergroup difference (p=0.147) (**Table 5**; **Figure 3**).

**Table 5:** Quality of life scale before and after AOM LAI (0=very bad, 1=bad, 2=neither good-nor bad, 3=good, 4=very good).

	Sample of patients					
	Patients in the community			Confined patients		
	Average	Typ. Deviation	N	Average	Typ. deviation	N
Quality of life - 6 months before AOM LAI	0.5	0.6	34	0.9	0.6	21
Quality of life - 6 months after AOM LAI	3.0	0.8	34	3.0	0.7	21

In blue, patients in the community, in yellow-orange, patients in the penitentiary system.

## Discussion

This study is the first in Greece to evaluate aripiprazole LAI for community patients with psychosis and SUD, and the first in Europe for incarcerated patients with unspecified psychosis and SUD. Observed outcomes may reflect temperamental traits, as noted by Favaretto et al. (2024) (9), which influence psychopathology severity and treatment engagement, especially in dual-diagnosis or Cluster B populations (10). Aripiprazole’s D2 partial agonism enhances prefrontal dopamine and GABA transmission, improving working memory (6) and social skills (7). It is indicated for schizophrenia relapse prevention, bipolar I manic episodes, and autism-related irritability and may benefit conduct disorders linked to AUD or cannabis use (11). A Madrid study (2020) reported a >30% CGI-S reduction and reduced substance use in 40 schizophrenia patients with SUD after six months of aripiprazole LAI (12). A German study (2018) highlighted cost savings, with hospitalization rates dropping from 55.1% to 14% post-switch to aripiprazole LAI (13).

Our findings confirm significant improvements in quality of life, functionality, and hospitalization rates, aligning with Sampogna et al. (2023) on LAI benefits (14). For incarcerated patients, 19 of 21 maintained stability, though two relapsed post-release, underscoring adherence challenges. The study supports expanded community mental health services, including crisis intervention and early psychosis programs, to reduce incarceration cycles.

Aripiprazole LAI also shows promise for AUD, with four community patients achieving abstinence and others reducing consumption. Animal studies support its efficacy in reducing ethanol-related behaviors (15, 16). Further research is needed to quantify cost savings and explore off-label use in AUD.

## Limitations

The study focused solely on aripiprazole LAI, limiting generalizability to other LAIs. The small sample size and potential self-report bias are additional constraints. In Greece, inadequate emergency psychiatric assessment may underestimate hospitalization needs, particularly for incarcerated patients with Cluster B traits and SUD.

## Data Availability

The datasets presented in this article are not readily available because The data is controlled exclusively by the main research team. Requests to access the datasets should be directed to epasparakis@agnhosp.gr.

## References

[1] Di Sciascio G Riva MA. Aripiprazole: from pharmacological profile to clinical use. Neuropsychiatr Dis Treat. (2015) 11:2635. doi: 10.2147/NDT.S88117, PMID: 26508859 PMC4610784

[2] ABILIFY (aripiprazole) tablets, ABILIFY DISCMELT (aripiprazole) orally disintegrating tablets, ABILIFY (aripiprazole) oral solution, ABILIFY (aripiprazole) injection for intramuscular use only. Otsuka Pharmaceutical Company (2014).

[3] Abilify 7.5 mg/ml solution for injection (intramuscular) – summary of product characteristics (SmPC). Available online at: https://www.medicines.org.uk/emc/product/6239/pil (Accessed January 20, 2023).

[4] DeLeon A Patel NC Crismon ML. Aripiprazole: a comprehensive review of its pharmacology, clinical efficacy, and tolerability. Clin Ther. (2004) 26:649–66. doi: 10.1016/S0149-2918(04)90066-5, PMID: 15220010

[5] Millan MJ. The neurobiology and control of anxious states. Prog Neurobiol. (2003) 70:83–244. doi: 10.1016/S0301-0082(03)00087-X, PMID: 12927745

[6] Murphy A Dursun S McKie S Elliott R Deakin JF. An investigation into aripiprazole’s partial D_2_ agonist effects within the dorsolateral prefrontal cortex during working memory in healthy volunteers. Psychopharmacol (Berl). (2016) 233:1415–26. doi: 10.1007/s00213-016-4234-9, PMID: 26900078 PMC4819596

[7] Lee JS Lee JD Park H-J Oh M-K Chun JW Kim S-J. Is the GABA system related to the social competence improvement effect of aripiprazole? An (18)F-fluoroflumazenil PET study. Psychiatry Investig. (2013) 10:75–80. doi: 10.4306/pi.2013.10.1.75, PMID: 23482902 PMC3590434

[8] Hahn M Roll SC. Dosing recommendations of aripiprazole depot with strong cytochrome P450 3A4 inhibitors: A relapse risk. Drug Saf Case Rep. (2016) 3:5. doi: 10.1007/s40800-016-0027-7, PMID: 27747685 PMC5005780

[9] Favaretto E Bedani F Brancati GE De Berardis D Giovannini S Scarcella L. Synthesising 30 years of clinical experience and scientific insight on affective temperaments in psychiatric disorders: State of the art. J Affect Disord. (2024) 362:406–415. doi: 10.1016/j.jad.2024.07.011, PMID: 38972642

[10] Burnette EM Nieto SJ Grodin EN Meredith LR Hurley B Miotto K. Novel agents for the pharmacological treatment of alcohol use disorder. Drugs. (2022) 82:251–74. doi: 10.1007/s40265-021-01670-3, PMID: 35133639 PMC8888464

[11] Coles AS Knezevic D George TP Correll CU Kane JM Castle D. Long-acting injectable antipsychotic treatment in schizophrenia and co- occurring substance use disorders: A systematic review. Front Psychiatry. (2021) 12:808002. doi: 10.3389/fpsyt.2021.808002, PMID: 34975600 PMC8715086

[12] Szerman N Basurte-Villamor I Vega P Martinez-Raga J Parro-Torres C Cambra Almerge J. Once-monthly long-acting injectable aripiprazole for the treatment of patients with schizophrenia and co-occurring substance use disorders: A multicentre, observational study. Drugs Real World Outcomes. (2020) 7:75–83. doi: 10.1007/s40801-020-00178-8, PMID: 32026379 PMC7060971

[13] Potempa C Rychlik R. Hospitalization rates and resource utilization of schizophrenic patients switched from oral antipsychotics to aripiprazole-depot in Germany. Health Econ Rev. (2018) 8:30. doi: 10.1186/s13561-018-0215-5, PMID: 30470936 PMC6755603

[14] Sampogna G Di Vincenzo M Giuliani L Menculini G Mancuso E Arsenio E. A systematic review on the effectiveness of antipsychotic drugs on the quality of life of patients with schizophrenia. Brain Sci. (2023) 13:1577. doi: 10.3390/brainsci13111577, PMID: 38002537 PMC10669728

[15] Ingman K Kupila J Hyytia P. Effects of aripiprazole on alcohol intake in an animal model of high-alcohol drinking. Alcohol. (2006) 41:391–8. doi: 10.1093/alcalc/agl037, PMID: 16684847

[16] Jerlhag E. The antipsychotic aripiprazole antagonizes the ethanol- and amphetamine- induced locomotor stimulation in mice. Alcohol. (2008) 42:123–7. doi: 10.1016/j.alcohol.2007.11.004, PMID: 18358991

